# Failure Mechanisms of an Al 6061 Alloy Foam under Dynamic Conditions

**DOI:** 10.3390/ma14061349

**Published:** 2021-03-11

**Authors:** Francesca Campana, Edoardo Mancini, Daniela Pilone, Marco Sasso

**Affiliations:** 1 DIMA, Sapienza Università di Roma, via Eudossiana 18, 00184 Roma, Italy; francesca.campana@uniroma1.it; 2 DIIIE, Università degli studi dell’Aquila, Piazzale Ernesto Pontieri, Monteluco di Roio, 67100 L’Aquila, Italy; edoardo.mancini@univaq.it; 3 DICMA, Sapienza Università di Roma, via Eudossiana 18, 00184 Roma, Italy; 4 DIISM, Università Politecnica delle Marche, Via Brecce Bianche, 60131 Ancona, Italy; m.sasso@staff.univpm.it

**Keywords:** aluminum foams, fracture behavior, failure mechanisms, Hopkinson bar

## Abstract

The interesting properties of Al 6061 aluminum foams have boosted the research on the correlation between foam composition and morphology and its mechanical response under dynamic conditions. In this study, ingots of an Al 6061-T4 foam were sectioned and analyzed in order to determine their microstructural and morphological characteristics, and then quasi-static and dynamic tests (10^−3^ to 3 × 10^2^ s^−1^) were carried out to determine the material mechanical behavior. Dynamic tests, carried out by using the split Hopkinson bar, highlighted that the studied foam is characterized by a very good energy absorption capability, due to its ductile behavior. Nevertheless, the conducted research showed that cell morphology and distribution affect its mechanical behavior in dynamic conditions in which localized cell collapse may result in a decreased energy absorption and efficiency of the foam.

## 1. Introduction

Porous materials have many interesting properties due to a unique combination of physical and mechanical characteristics. Over the last decades, metallic foams have been deeply studied and different applications have been considered. Depending on the selected alloy and pore morphology, metallic foams can be of great interest for functional and structural applications [1,2,3]. Among them, one of the most remarkable applications is related to energy absorption because metallic foams combine strength and the capability of deforming at nearly constant stress levels. In order to comply with passive safety regulations, metallic foam components can be usefully designed, but this needs a deep investigation on the dynamic response of cellular material up to the densification stage.

Many papers concerning the behavior of metallic foams under impact loading are available in the literature [4,5,6,7,8], and many of them concern studies carried out by using the Hopkinson bar that allows performing an accurate evaluation of the material response [4,5,7,9]. Nowadays, the Split Hopkinson Bar (SHB) represents the most established technique to perform tension, compression, and even torsion tests on engineering materials in the strain rate range from 10^2^ s^−1^ to 10^4^ s^−1^; it has been extensively used on metals [10,11] and construction materials [12], on polymers and composites [13], and biomedical materials [14]. The research in [7] studied the effects of the strain rate and inertia on the deformation behavior of closed-cell aluminum foams under impact, highlighting that three different deformation modes can be identified—homogeneous mode, transitional mode, and shock mode. It has been revealed that the axial-inertia effect becomes more relevant than the strain rate effect when high-speed impact occurs. These findings can probably explain the contradictory results available in the literature about the aluminum foam sensitivity to the strain rate.

The energy absorption capability of foams originates from material permanent deformation and then from deformation and fracture modes of cell walls. Then, the mechanical behavior of metallic foams depends on several factors such as type of alloy, relative density, cell distribution, and morphology. Alloy composition and possible heat treatment affect both energy absorption capability and fracture toughness of the alloy [15,16]. In the literature, the relationship between relative density and mechanical properties of aluminum foams has been also investigated [17,18,19,20,21] and a linear correlation has been found by some authors. Despite these considerations, cell morphology and inhomogeneity of the foam structure are considered the primary factors determining the deformation modes. Studies available in literature highlighted four failure modes of closed-cell aluminum foam, showed energy absorption mechanisms, and revealed also that the friction between cells during compaction further increases the energy absorption [20,21,22]. On the other hand, cell size, shape, and distribution depend on the used production process parameters; for this reason, some design tools have been studied for analyzing the correlation between process parameters, cell distribution, and morphology with the aim of tailoring the production process on a specific material application [23,24,25].

In-vehicle crashworthiness design aluminum alloy foams are usefully used for passive safety. In order to promote their use, it is of paramount importance to study the relationship between alloy composition, cell morphology, and foam mechanical behavior under dynamic conditions. Among aluminum alloys, Al 7075 and Al 6061 alloys seem to be very interesting because their mechanical properties can be tailored to a specific application by selecting heat treatment parameters. In a previous study, the authors presented the dynamic characterization of an Al 7075 closed-cell material and demonstrated, by using a 3D model, that cell morphology determines a scattering of the results [26,27].

In this study, the authors studied the mechanical behavior of an Al 6061 alloy foam under dynamic conditions with the aim of studying how the base material composition and the pore morphology affect the mechanical response.

## 2. Materials and Methods

### 2.1. Foam Morphological and Microstructural Characterization

The Al 6061-based foams investigated in this research were supplied by IFAM in Bremen, Germany. They were manufactured by using as foaming agent a titanium hydride that decomposes at about 465 °C, producing gas release and then foam formation. The foam ingots have a square base section (45 mm × 45 mm) and 100 mm height. The research was carried out by using the following methodologies: image analysis, used for studying cell distribution inside the specimen, and mechanical characterization carried out by crushing. All foam specimens were characterized in terms of density using a caliper and a microbalance. The nominal composition of the alloy is reported in Table 1.

**Figure 1:**
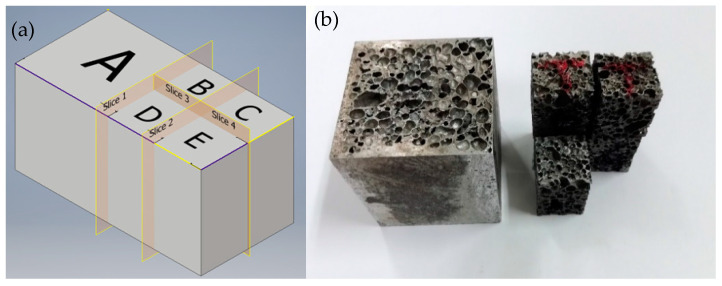
(**a**) Scheme of the ingot sectioning and (**b**) macrograph of the ingot after sectioning.

The ingots were sectioned following the scheme reported in Figure 1. The specimens for the mechanical tests were obtained from block A, while blocks B, C, D, and E were used for performing image analysis and thus for carrying out a morphological characterization of ingots on slices 1, 2, 3, and 4. This choice has been made since the ingots seem to have a symmetrical cell distribution in the longitudinal direction (slice 1 can be considered the plane of symmetry).

Samples were cut minimizing cell wall damage. The surfaces were painted using a black dye in order to improve the contrast that must be high for performing image analysis. The specimens were ground by using SiC papers and polished with 1 μm and 0.3 μm alumina suspension. Cell size, roundness, and mean area were determined, for every studied slice, by using Leica LAS software (Leica, Wetzlar, Germany).

Microstructural examination of polished cross sections was performed, after etching with the Keller’s reagent, by using an optical microscope (Leica DMI 5000, Leica, Wetzlar, Germany), while fracture surfaces were inspected by using a scanning electron microscope (SEM) (Hitachi, Tokyo, Japan). Compositional analyses were performed using energy dispersion spectroscopy (EDS) (Noran System Six, Thermoscientific, Madison, WI, USA).

The characteristics of the samples, which were cut from block A and tested in quasi-static (QS) and dynamic (DYN) conditions, are reported in Table 2 in terms of specimen size (a × b × h) and effective density.

### 2.2. Quasi-Static and Dynamic Tests

**Figure 2:**
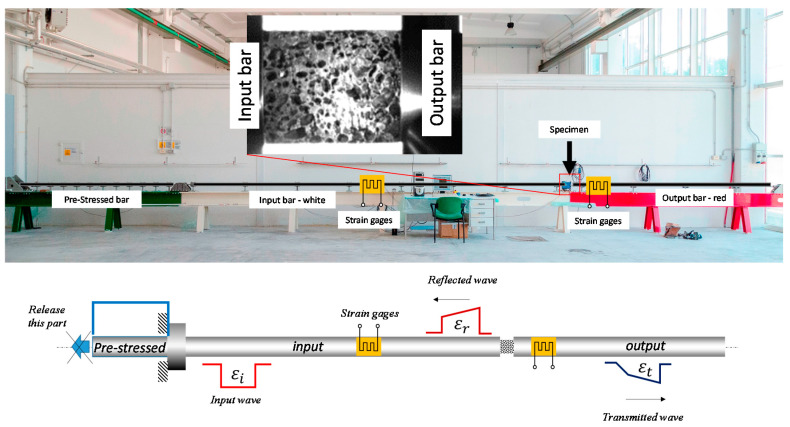
Hopkinson bar facility image and scheme.

Quasi-static compression tests were performed by using an electro-mechanical tensile machine (Zwick/Roell Z050, Genova, Italy), while dynamic compression tests were performed by using the self-designed SHB facility installed at Università Politecnica delle Marche (Ancona, Italy) [29,30] (Figure 2). The quasi-static tests were conducted at a strain rate of 10^−3^ s^−1^, whereas dynamic tests were conducted at approximately 3·10^2^ s^−1^. In both types of tests, the specimen surfaces were lubricated by using MoS_2_ grease (Molykote^®^ BR-2). It is worth noting that higher strain rates, up to several thousand s^−1^, are usually reached in SHB tests on bulk materials. However, in the present case, the specimen cross section and length were necessarily larger than usual in order to make the sample sufficiently representative of the heterogeneous material; this explains the relatively low strain rate achieved in the SHB tests.

The used SHB consists of three aligned bars, respectively named pre-tensioned, input, and output bar, having a length of 3.0 m, 7.5 m, and 4.0 m. All bars were made of 17-4 PH steel with a diameter of 18 mm. An electromechanical actuator pulls the pre-tensioned bar at one end, while the bar is stopped against a rigid block at the other end. The tensile load is suddenly released by the failure of the sacrificial brittle element connecting the actuator and the pre-tensioned bar; the latter keeps moving forward and hits the input bar. The impact generates a compression wave that travels along the input bar at sound speed, *C*_0_. The input wave reaches the specimen, which is sandwiched between the input and the output bar. In this phase, the specimen is quickly deformed, and the stress wave is partially reflected back in the input bar and partially transmitted to the output bar. The setup is designed so that the bars remain within their elastic limit, while the specimen undergoes large deformations; indeed, even if the achieved strain rates are not very high, the length of the input wave is enough to deform the foam specimens up to almost 40% of strain.

The stress and strain experienced by the samples are computed by post-processing the signals measured by strain gauges appropriately placed on the input and output bars. The strain signals are recorded at 1 MHz by means of a NI PCI 6120 acquisition card. Denoting with *ε_R_* and *ε_T_* the reflected and transmitted strain waves, in the hypotheses of specimen equilibrium, the desired engineering strain and stress in the sample are retrieved by the classical Hopkinson Bar formulas as follows:

(1) $$\varepsilon \left(t\right)=-2\frac{C_{0}}{L_{S}}\int_{0}^{t}\varepsilon_{R}\left(t\right)dt$$

(2) $$\sigma \left(t\right)=E\frac{A_{b}}{A_{S}}\varepsilon_{T}\left(t\right)$$

where *L_S_* and *A_S_* are the initial length and cross-section area of the sample, and *E* and *A_b_* are the elastic modulus and cross-section area of the bars.

## 3. Results and Discussion

**Figure 3:**
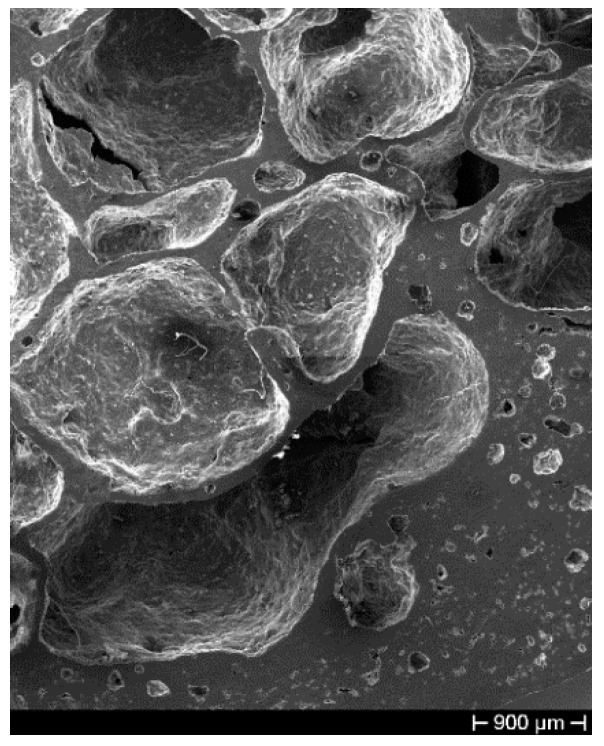
Scanning electron microscope (SEM) micrograph showing pore morphology.

Samples of the Al 6061 alloy foam have been analyzed by using an optical and scanning electron microscope. The SEM micrograph at low magnification in Figure 3 shows the morphology of the cavities—as is clearly visible in the figure, cavities have a very irregular shape and some of them come from the merger of two or more voids. Moreover, a considerable number of very small cavities are visible in correspondence of the foam walls; their origin is the foaming process and shrinkage during solidification. Their presence in the bulk material constituting the cell walls could strongly affect the foam mechanical behavior.

**Figure 4:**
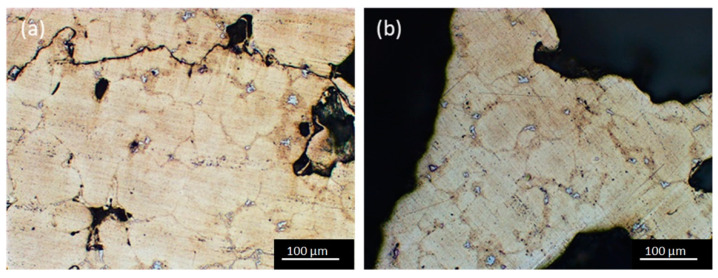
Optical micrograph showing the microstructure of (**a**) the alloy of the external wall and (**b**) the wall between adjacent cavities.

**Figure 5:**
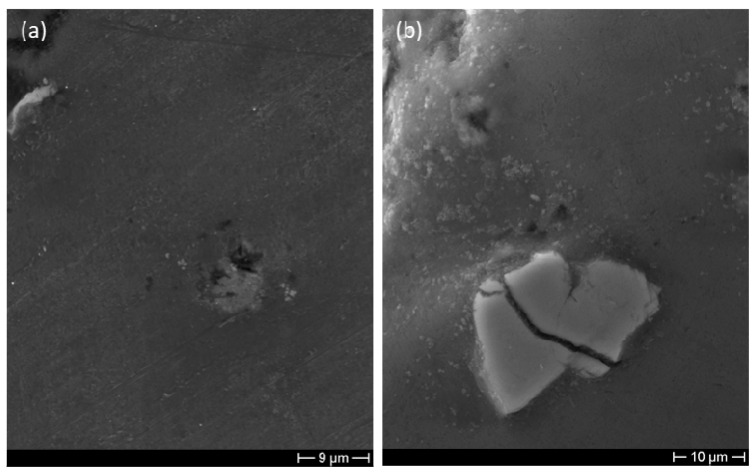
SEM micrographs showing (**a**) an Fe-based intermetallic particle and (**b**) a Ti particle.

In order to analyze the alloy microstructure, specimens after grinding and polishing have been studied by using the optical microscope. Micrographs in Figure 4 show that the alloy microstructure is characterized by α grains surrounded by the eutectic phase. In correspondence with the grain boundaries, the presence of irregular particles (bright phase in Figure 4) can be noticed. These particles have been analyzed by energy dispersion spectroscopy (EDS). The analyses highlighted that some of them are Fe-based intermetallic particles as shown in the literature [31,32], while others are titanium particles deriving from the foaming process. In fact, when TiH_2_ decomposes, while hydrogen gas determines the formation of cavities, Ti particles remain embedded in the alloy. Figure 5 shows in detail the two types of particles. Microhardness tests carried out on the alloy showed an average value of 77 HV_0.05_, although the grain boundary hardness reached about 92 HV_0.05_ due to the presence of intermetallic particles. The mean hardness value suggests that the alloy has been subjected to natural aging (T4 temper state). The presence in the bulk alloy of shrinkage cavities, intermetallic particles, Ti particles, and cracks (Figure 4a) suggests that they could produce initiation and propagation of cracks when a load is applied.

**Figure 6:**
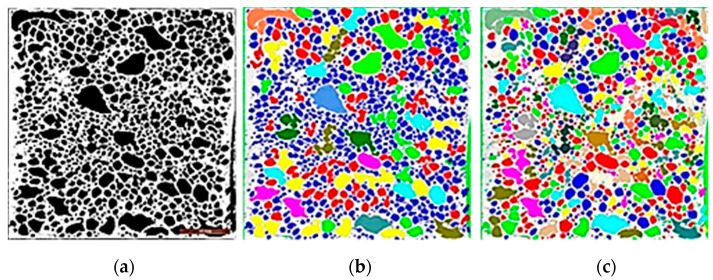
Segmentation of (**a**) a foam image to analyze (**b**) size and (**c**) roundness of cells.

Considering that the mechanical behavior of the aluminum foam depends on the alloy microstructure and the size and distribution of cavities, it is very important to characterize cell morphology. After the acquisition of the images of the different sections of the ingots, images have been pre-processed to remove noise, then segmentation has been performed to identify the regions of interest. In this way, features such as cell size and roundness have been studied (Figure 6).

**Figure 7:**
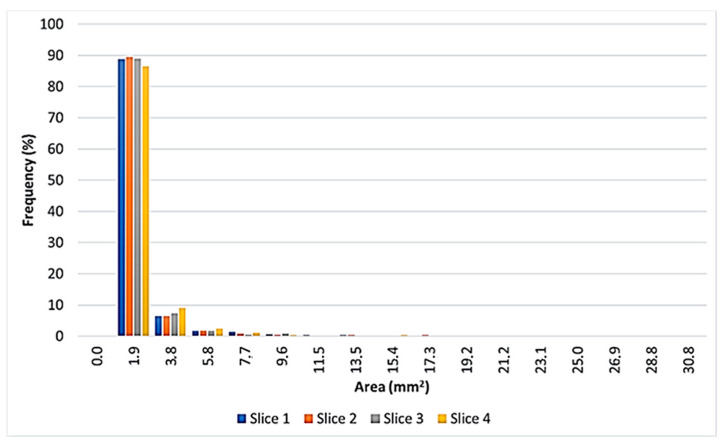
Distribution of cell size.

Figure 7 highlights that about 90% of cells have an area lower than 1.9 mm^2^, while the highest number of cavities, with an area between 1.9 mm^2^ and 3.8 mm^2^, is located close to the ingot ends. The analysis of the data reveals also that in every slice it is possible to find few big cavities.

**Figure 8:**
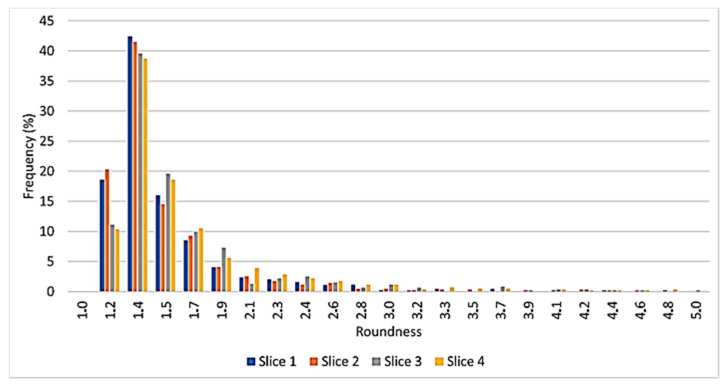
Distribution of cell roundness.

Figure 8 shows the cell roundness distribution. Cell roundness, *R*, is an adimensional value defined by

(3) $$R=\frac{L^{2}}{4\pi A_{i}\cdot 1.064}$$

where *L* is the cell perimeter, *A_i_* is for *i*_th_ void area, and 1.064 is a compensation factor that takes into account the error introduced in the area calculation by the digitalization of the image in which continuous perimeters are approximated by discrete rectangles. Considering that a circle has a roundness equal to 1, the most rounded cavities are visible in the ingot transversal sections. Cells seem to be more elongated in the longitudinal direction. This is probably due to the orientation of the mold in the foaming stage. Moreover, a close examination of Figure 8 highlights that in all considered slices, there are few very irregular cavities that may become stress concentration sites. On the ground of these results, it is possible to suppose that the foam behavior will be affected by load direction.

**Figure 9:**
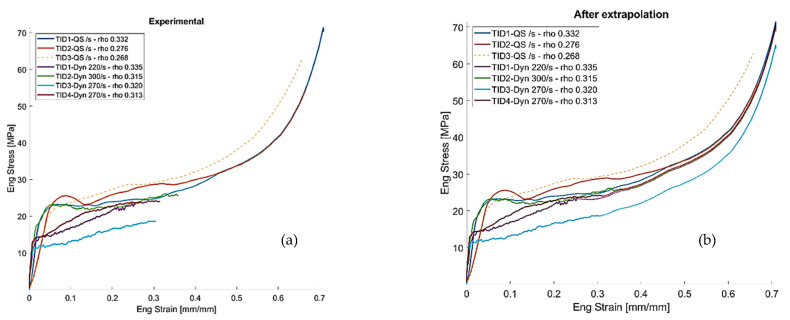
Stress–strain curves obtained in quasi-static conditions and at different strain rates (**a**) before and (**b**) after extrapolation.

Stress–strain curves obtained for strain rates varying over the range 10^−3^ s^−1^ to 220–300 s^−1^ are useful to understand foam behavior. Figure 9 shows the results of four tests carried out on specimens having a relative density varying from 0.31 to 0.33, in which the relative density is the ratio between the effective density of the tested specimen divided by the aluminum density. Papers available in the literature show that the Al 6061 alloy is not sensitive to strain rate in the investigated range [33,34]. By observing the curves reported in Figure 9, it is evident that there is not a direct correlation between strain rate and curve trends and that all curves tend to move close to the quasi-static curves. This behavior can be ascribed to the presence of irregular cell shapes and distribution that affect the material behavior.

Dynamic curves, due to limitations related to maximum stroke available, are shorter than the quasi-static ones; for this reason, they have been extrapolated by the experiments (Figure 9b) for the subsequent efficiency evaluation.

**Figure 10:**
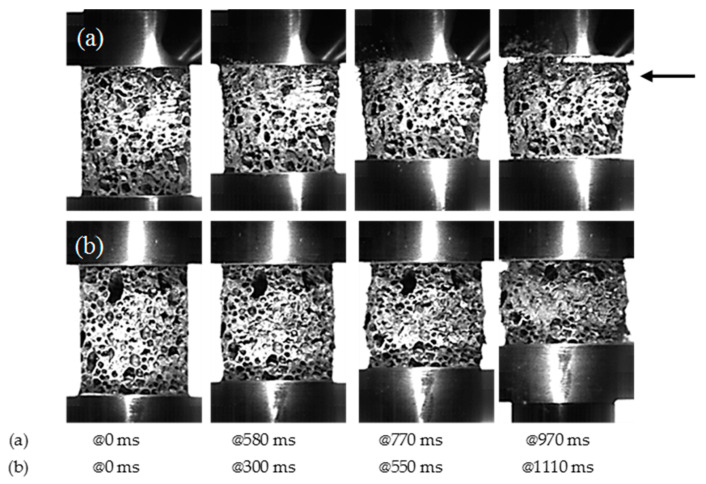
Frames of the video recorded during (**a**) TID3-Dyn and (**b**) TID2-Dyn tests.

The absence of an initial peak and the curve smooth trends suggest that the cell walls bend during the tests without fracturing and hence that the material has a ductile behavior. A close observation of the foam behavior during the dynamic tests indicates that the specimen mechanical response is closely related to the cell distribution. Figure 10 shows some frames of the video recorded during the TID3-Dyn (a) and the TID2-Dyn (b) tests. It can be noticed that in contrast to Figure 10a in which the deformation is localized in an area where the material could not bear the stress because of cell morphology, in Figure 10b, cell wall deformation occurs through all the specimens. This suggests that the presence in the material of a weak region causes localized cell collapse and a decrease of the material strength that increases only when adjacent areas will resist the applied stress. When cell wall deformation occurs in all the specimen volume, the stress–strain curve obtained in dynamic condition overlap with the one obtained in quasi-static conditions.

**Figure 11:**
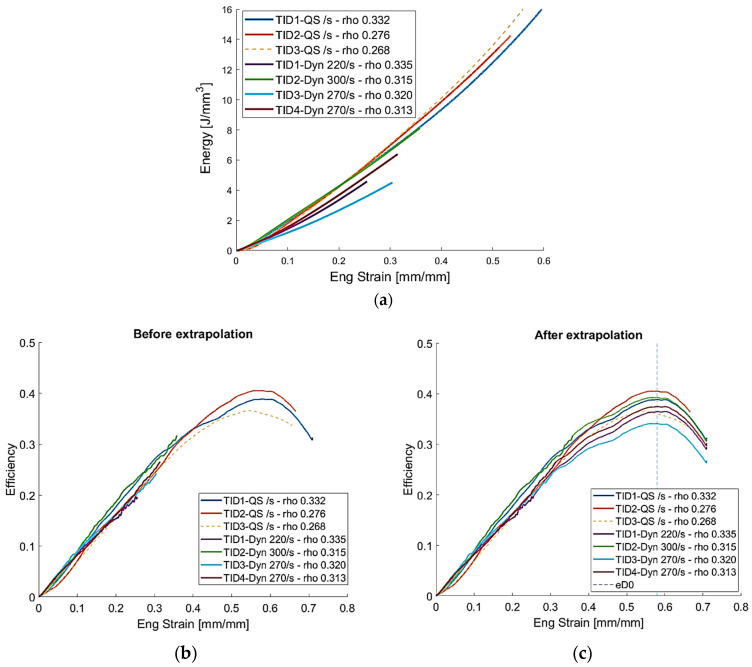
(**a**) Foam energy absorption and (**b**) efficiency before and (**c**) after extrapolation.

Figure 11 shows the foam energy absorption and efficiency. The energy absorption efficiency was evaluated as proposed in [35,36]. The localized cell collapse affects the energy absorption (Figure 11a) and the foaming efficiency (Figure 11b,c); in fact tests with localized deformation are characterized by a reduced efficiency value. By analyzing the curves, it is evident that the adoption of these foams in energy absorber elements to improve vehicle crashworthiness during high-speed impact would be beneficial only if there is the possibility of setting up a production process that allows close control of size, shape, and distribution of cells.

**Figure 12:**
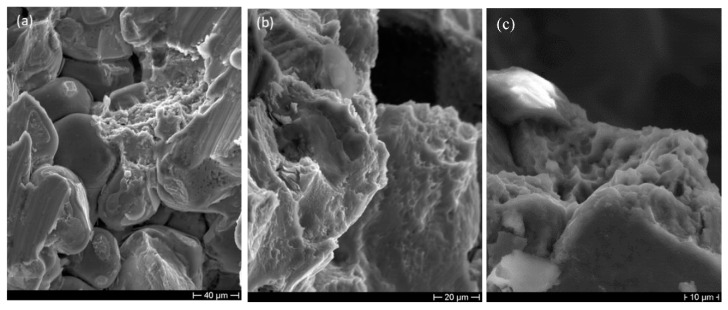
SEM micrographs showing the fracture surfaces close to (**a**) a shrinkage cavity and (**b**,**c**) in the defect-free area where dimpled surfaces are evident.

Important information about the fracture mechanism can be obtained by observing the fracture surfaces. SEM analyses highlight that the fracture of cell walls is predominantly ductile; in fact Figure 12b,c highlight the presence of dimples and of considerable plastic deformation. In some areas, it is possible to observe a mixed-mode fracture probably due to the presence of very irregular micro-shrinkage cavities (Figure 12a) and intermetallic particles that may locally lower the fracture toughness of the alloy.

## 4. Conclusions

The tests carried out in this research highlighted that the Al 6061 alloy foam behavior under dynamic conditions is considerably affected by cell distribution and morphology. Image analysis revealed that cells seem to be more elongated in the longitudinal direction, and this is probably due to the orientation of the mold in the foaming stage. Moreover, it revealed that in all the considered slices there are few very irregular cavities that may affect foam behavior. The obtained results revealed that the presence of big and irregular cells could determine a localized material collapse with consequent efficiency and energy absorption decrease. Although fractographic examinations revealed that the alloy behavior is predominantly ductile, the presence of Fe-rich intermetallics and shrinkage cavities could make the cell walls more brittle with consequent worsening of the foam behavior reliability. On the ground of these results, it seems advantageous to use the studied foam for the development of energy absorber elements to improve vehicle crashworthiness during low-speed impact.

## Figures and Tables

**Table 1:** The nominal composition of Al 6061 alloy (wt.%) [28].

Mg	Si	Cu	Cr	Mn	Fe	Al
0.8–1.2	0.4–0.8	0.15–0.4	0.04–0.35	0–0.15	0–0.7	Bal.

**Table 2:** Characteristics of the samples used for quasi-static (QS) and dynamic (DYN) tests.

TID#	Type	a	b	Length (h)	ρeff
		(mm)	(mm)	(mm)	(kg/m^3^)
1	QS	17.93	19.55	21.27	895
2	QS	18.46	19.90	20.56	746
3	QS	17.92	18.28	14.80	723
1	Dyn	17.95	19.22	21.96	904
2	Dyn	17.84	18.41	21.02	852
3	Dyn	18.16	19.15	21.77	865
4	Dyn	18.29	18.35	21.60	846
				Mean	833
				Std. dev.	70.8

## Data Availability

Data is contained within the article.
